# The Neurobiological Underpinnings of Psychoanalytic Theory and Therapy

**DOI:** 10.3389/fnbeh.2018.00294

**Published:** 2018-12-04

**Authors:** Mark Leonard Solms

**Affiliations:** Psychology, University of Cape Town, Cape Town, South Africa

**Keywords:** psychoanalysis, neurobiology, basic emotions, unconscious, repression, efficacy

## Abstract

This paper sets out the neurobiological underpinnings of the core theoretical claims of psychoanalysis. These claims concern (1) innate emotional needs, (2) learning from experience, and (3) unconscious mental processing. The paper also considers the neurobiological underpinnings of the mechanisms of psychoanalytic treatment—a treatment which is based on the aforementioned claims. Lastly, it reviews the available empirical evidence concerning the therapeutic efficacy of this form of treatment.

I recently published a short article in the *British Journal of Psychiatry* (international edition; Solms, 2018a) concerning the scientific standing of psychoanalysis. Implicit in that article were numerous neurobiological assumptions and hypotheses, which I would like to unpack here. This article also builds upon two other partial attempts to explicate these hypotheses (Solms, 2017b; Smith and Solms, 2018), in the *Annals of the New York Academy of Sciences* and *Neuropsychoanalysis*, respectively. There is some overlap between the present article and these previous articles, but the present effort attempts to go further and reveal an overarching picture.

My aim in the first article mentioned above was to set out what psychoanalysts may consider to be *the core scientific claims of their discipline*. Such scientific stock-taking is necessary at this stage in the history of psychoanalysis, due to widespread misconceptions among the public and neighboring disciplines, and disagreements among psychoanalysts themselves regarding specialist details, which obscure a bigger picture upon which most of us can agree.

I addressed three questions in the first article cited above (Solms, 2018a), namely: (A) How does the emotional mind work, in health and disease? (B) On this basis, what does psychoanalytic treatment aim to achieve? (C) How effective is it? My arguments in relation to these questions were:

- Psychoanalysis rests upon three core claims about the emotional mind that were once considered controversial but which are now widely accepted in neighboring disciplines (here, I am referring principally to neurobiology).
- The clinical methods that psychoanalysts use to relieve mental suffering flow directly from these core claims, and are consistent with current scientific understanding of how the brain changes.
- It is therefore not surprising that psychoanalytic therapy achieves good outcomes—at least as good as, and in some important respects better than, other evidence-based treatments in psychiatry today.

Now I will unpack these arguments, spelling out the neurobiological underpinnings which were partially explicated in the other two articles cited above (Solms, 2017b; Smith and Solms, 2018). These underpinnings pertain especially to the first argument, much less so to the second, and least to the third. This is because questions about how and whether psychoanalytic therapy works are necessarily predicated upon claims about how the emotional mind works. The three sections of this article will, accordingly, be of unequal length.

I submit that the core claims of psychoanalysis regarding the emotional mind are the following:

1. The human infant is not a blank slate; *like all other species, we are born with a set of innate needs*.
2. *The main task of mental development is to learn how to meet these needs in the world*, which implies that mental disorder arises from failures to achieve this task.
3. *Most of our methods of meeting our emotional needs are executed unconsciously*, which requires us to return them to consciousness in order to change them.

These core claims could also be described as foundational *premises*, but it is important to recognize that they are *scientific* premises, because they are testable and falsifiable. As I proceed, I will elaborate the core claims, adding details, but I want to distinguish between the core claims themselves and the specifying details. The details are empirical. Whether they are ultimately upheld or not does not affect the premises. Detailed knowledge develops over time, but premises are foundational.

For example, by analogy: a core claim of evolutionary biology is that species evolve by means of natural selection (Darwin, 1859). If this claim were disproven, then the whole theory of evolution would be rejected. With the early twentieth century integration into evolutionary theory of Mendel's laws of inheritance—about which Darwin knew nothing—the modern science of genetics was established. The same applied to the mid twentieth century discovery of DNA—the actual medium of inheritance, about which Darwin likewise had no inkling. This established the modern science of molecular biology. Molecular biology in turn led to the discovery in the late twentieth century of epigenetic regulatory programmes, revealing a whole new domain called evolutionary developmental biology—some of the findings of which directly contradict aspects of Darwin's thinking. All of these developments have elaborated the empirical *contents* of evolutionary theory—they have not shaken its *foundations*.

The same applies to psychoanalysis. Everything psychoanalysts do is predicated upon the above three claims. If *they* are disproven, the core scientific presuppositions upon which psychoanalysis (as we know it) rests will have been rejected. But as things stand currently, they are eminently defensible, supported by accumulating and converging lines of evidence in neurobiology. This justifies the assertion that “Psychoanalysis still represents the most coherent and intellectually satisfying view of the mind (Kandel, 1999).” However, in this article, I will also draw attention to some crucial errors in the contents (as opposed to foundations) of Freud's classical conception of the mind.

I turn now to the three identified core claims.

## Claim 1

*The human infant is not a blank slate; like all other species, we are born with a set of innate needs*.^1^ The innate needs of the human organism are regulated autonomically up to a point. But beyond that point they make “demands upon the mind to perform work,” as Freud (1915a) put it. Once bodily demands become mental, they constitute what Freud called the “id.”

Freud recognized that drive demands are ultimately felt as affects. This fact alone (i.e., the fact that the *fundamental needs of the organism* are felt in the pleasure-unpleasure series) explains why affect is so important in psychoanalysis (cf. Freud's “pleasure principle”). But what Freud did not realize is that such demands are actually felt at their *source*. In other words, there is evidence to suggest that drives, which Freud (1905) located at “the frontier between the mental and the somatic” become mental *when they are felt*, prior to which they are not drives but rather autonomic regulatory mechanisms (for summaries of this evidence, see Panksepp, 1998; Solms, 2013; Damasio, 2018).

Freud imagined that id demands take the form of unconscious drive “energies” which operate within the mind and only become conscious when they are registered by the superficial “system Pcpt-Cs,” which he located in the cerebral cortex.^2^ The mistaken assumption underlying this theory, namely that consciousness is an intrinsic property of cortex, was first revealed in the 1940s, i.e., shortly after Freud died. The critical experiments were performed by Moruzzi and Magoun (1949), who showed that consciousness in cats is generated not in the cortex but rather in the upper brainstem, in a region now known as the “extended reticulothalamic activating system” (ERTAS). Confirmation that the same applied to humans was quickly forthcoming, for example from Penfield and Jasper (1954), who observed that consciousness is only lost during seizures when epileptogenic activity spreads to what they called the “centrencephalic” region. These observations have stood the test of time, although the role (in the generation of consciousness) of some non-ERTAS upper-brainstem structures, such as the PAG, and even higher (limbic) circuits, has gradually been recognized (Panksepp, 1998; Merker, 2007).

The whole situation I am addressing is summed up in the following statement by Freud (1920, p. 24)—who, incidentally, started his scientific life as a neuroanatomist:

What consciousness yields consists essentially of perceptions of excitations coming from the external world and of feelings of pleasure and unpleasure which can only arise from within the mental apparatus; it is therefore possible to assign to the system *Pcpt.-Cs*. a position in space. It must lie on the borderline between outside and inside; it must be turned toward the external world and must envelop the other psychical systems. It will be seen that there is nothing daringly new in these assumptions; *we have merely adopted the views on localization held by cerebral anatomy, which locates the “seat” of consciousness in the cerebral cortex*—the outermost, enveloping layer of the central organ. Cerebral anatomy has no need to consider why, speaking anatomically, consciousness should be lodged on the surface of the brain instead of being safely housed somewhere in its inmost interior (emphasis added).

Ironically, it turns out that consciousness *is* lodged in the brain's inmost interior. Consciousness is an *endogenous* property of the brain; it does not stream in through the senses.

The full implications of this discovery were slow to emerge, and they are only now being fully digested (see Panksepp et al., 2017). Initially, Moruzzi and Magoun—and just about everybody else—tried to save the old theory by drawing a distinction between the “contents” of consciousness (which they assigned to the cortex) and its “level” (which they assigned to the ERTAS). The so-called level of consciousness (or “wakefulness”) was therefore measured *quantitatively*—on a 15-point scale—while its (perceptual and cognitive) contents were assessed *qualitatively*. But evidence that “arousal” possesses qualities of its own is easily demonstrated. The supposed “level” of consciousness really consists in a variety of *states* of consciousness (cf. Mesulam, 2000). It *feels like something* to be awake. That is why the ERTAS and PAG are not a concern of anesthetists alone (or of neurosurgeons alone); they are of equal concern to psychiatrists. The neuromodulatory systems that are the targets of the best known psychoactive medications have their source cells in the ERTAS (Consider for example, serotonin, noradrenaline and dopamine.). Thus, it turns out that the contents of consciousness do not consist only in the sensory qualia of our classical exteroceptive modalities; the ERTAS generates endogenous qualia of its own. These contents or qualia are known as *affects*.

To be sure, affect is a *more fundamental* form of consciousness than the cortical form of it which attaches to the classical sensory modalities. The relationship between the two forms is hierarchical: *cortical consciousness (conscious perception and cognition) is dependent upon ERTAS arousal*. Thus, whereas even a small amount of damage to the ERTAS causes coma (Parvizi and Damasio, 2003), damage to large swathes of cortex results merely in a loss of “certain forms of information” (Merker, 2007, p. 65). The smallest area of brain tissue whose destruction causes total loss of consciousness is located just below the PAG, stimulation of which—importantly— produces the most extreme states of affective arousal known to man (both pleasurable and unpleasurable, depending on the precise site which is stimulated; see Panksepp, 1998; Merker, 2007).

That is why decorticate animals are conscious (Huston and Borbely, 1974), as are children born without cortex (Shewmon et al., 1999). These animals and children are totally devoid of cortical representations, yet they are awake and alert and display a wide range of emotional responses to adequate stimuli. This decisively contradicts the notion that emotions only become conscious if they are registered in (prefrontal or insular) cortex (cf. LeDoux, 1999; Craig, 2012). There is absolutely no evidence for this. In fact, decorticate animals are *excessively* emotional (Huston and Borbely, 1974), as are human beings with damaged prefrontal lobes (Harlow, 1868). Preserved—indeed enhanced—emotional consciousness can likewise be demonstrated in patients whose insular cortex is totally destroyed (Damasio et al., 2013).

But Freud shared the cortico-centric view of emotion. Thus, he (Freud, 1940, pp. 161–2) wrote:

The process of something becoming conscious is above all linked with the perceptions which our sense organs receive from the external world. From the topographical point of view, therefore, it is a phenomenon which takes place in the outermost cortex of the ego. It is true that we also receive conscious information from the inside of the body—the feelings, which actually exercise a more peremptory influence on our mental life than external perceptions; moreover, in certain circumstances the sense organs themselves transmit feelings, sensations of pain, in addition to the perceptions specific to them. Since, however, these sensations (as we call them in contrast to conscious perceptions) also emanate from the terminal organs and since *we regard all these as prolongations or offshoots of the cortical layer*, we are still able to maintain the assertion made above [at the beginning of this paragraph]. The only distinction would be that, as regards the terminal organs of sensation and feeling, the body itself would take the place of the external world (emphasis added).

So, for Freud, affects were only felt once they were “read out” in cortex, even though there was no evidence for the view that they are transmitted from terminal organs in the interior of the body to cortex via “prolongations or offshoots of the cortical layer.”^3^ There is, however, growing support for the view that affects emanate from the visceral interior of the body (see Damasio, 1994, 2018). Freud thought that affects register “oscillations in the tensions of drive needs” (1940, p. 198), and he defined “drive” as “the psychical representative of the stimuli originating from within the organism and reaching the mind, as a measure of the demand made upon the mind for work in consequence of its connection with the body” (Freud, 1915a, p. 122). In other words, *bodily “demands made upon the mind for work” are felt as affects*. On this basis, Damasio wrote that “Freud's insights on the nature of affect are consonant with the most advanced contemporary neuroscience views” (1999, p. 38).

It is certainly true that arousal states are *felt*; and many states of arousal are generated by drive needs. In short, *we become aware of our needs via feelings*. Consider hunger and thirst, for example. According to Damasio (1994), that is what feelings are *for*—which implies that is what *consciousness* is for, in its most basic form (Damasio, 2010, 2018). Affect is a value system, in terms of which pleasurable feelings signal states of the body that enhance the chances of survival and reproductive success, and unpleasurable feelings signal the opposite.

Significantly, as I have stated already, the mechanisms underpinning this—the most fundamental form of consciousness—are located in the upper brainstem and diencephalon. There, bodily “need detectors” (located principally but not exclusively in the medial hypothalamus) activate the basic arousal states that Panksepp (1998) calls “homeostatic affects.”

But there are also more complex types of affect, the source cells and circuits for which are located slightly higher in the brain. These “emotional” affects (such as fear and attachment bonding) and “sensory” affects (such as surprise and disgust) are no less crucial for survival and reproductive success than the homeostatic ones; but they do not simply register the current state of the body. These circuits, which release complex behavioral stereotypes like grooming, fighting, and copulating (and the feelings associated with them), are intrinsic to the brain itself. (This transcends the James-Lange theory of emotion). Emotional circuits, too, arise mainly in the upper brainstem but they also extend higher into the limbic system (see Panksepp, 1998). A useful way of distinguishing the types of affect—following Panksepp—is to differentiate between three broad levels: drives (*homeostatic* affects), instincts (*emotional* affects), and reflexes (*sensory* affects).

The important thing for present purposes, however, is this: all three types of affect are generated by the brain mechanisms which perform the functions that Freud assigned to the id—see Solms (2013) for detailed evidence—and they are all *conscious*. In fact, Freud himself always insisted that the notion of unconscious affect was an oxymoron (thereby contradicting his own theory that the id is simultaneously unconscious *and* regulated by the pleasure principle).

To sum up so far: consciousness registers the state of the *subject*, not (in the first instance) of the object world. The sentient subject is first and foremost an *affective* subject. Only then can we (consciously) experience perceptual and cognitive representations. That is why—to state the obvious—there can be no objects of consciousness without a subject of consciousness “being there” to experience them. The subject of consciousness is primary. The secondary (perceptual and cognitive) form of consciousness is achieved only when the subject of consciousness *feels* its way into its perceptions and cognitions, which are unconscious in themselves. The pseudopodia of an amoeba, palpating the world, come to mind (see Solms, 2017a for the empirical details behind these arguments).^4^

However, this is not the place to rehearse all the arguments in favor of the view that affects are felt at their source, in the upper brainstem, diencephalon, and limbic system. I have repeatedly summarized the evidence for this view elsewhere (e.g., Solms, 2013, 2017a,b; Solms and Friston, 2018). Such questions are not what matter most in the present context, where I am laying out the *core claims* of psychoanalysis. The core claim in this respect remains: The human infant is not a blank slate; like all other species, we are born with a set of innate needs, *and these needs are (ultimately) felt as affects*. Few neurobiologists today would dispute this core claim.

Now we can move on. Each affect which promotes—i.e., broadcasts the presence of—a need releases driven or instinctive or reflexive *behaviors*. These innate behavioral tendencies—of which there are a great many—consist in hard-wired *predictions* (i.e., stereotyped action plans; I am following Friston's terminology here; see Friston, 2010). Both Panksepp and LeDoux conceptualize these action tendencies as hereditary “tools for survival” (and therefore, of course, by extension, for reproductive success). In short, we execute these actions because they are designed to meet our (inescapable) biological needs—e.g., we cry, search, freeze, flee, attack, copulate.

These two concepts—innate *needs* and their associated *predictions*—underpin everything else I am going to say in this section.

Universal agreement about the number of such needs (and the associated innate behavioral predictions) in the human brain has not been achieved,^5^ but most mainstream taxonomies include at least a subset of the following *emotional* ones:

- We need to engage with the world—since all our biological appetites (including bodily needs like hunger and thirst) can only be met there.^6^ This is a *foraging* or seeking instinct. It is felt as interest, curiosity and the like. (It coincides roughly but not completely with Freud's concept of “libido;” see Solms, 2012).
- We need to find sexual partners. This is felt as *lust*. This instinct is sexually dimorphic (on average) but male and female inclinations exist in both genders. (Like all other biological appetites, lust is channeled through seeking)^7^.
- We need to escape dangerous situations. This is *fear*.^8^
- We need to attack and get rid of frustrating objects (things that come between us and satisfaction of our needs). This is *rage*.
- We need to attach to caregivers (those who look after us). Separation from attachment figures is felt not as fear but as *panic*, and loss of them is felt as *despair*. (The whole of “attachment theory” relates to this need, and the next one).^9^
- We need to care for and *nurture* others, especially our offspring. This is the so-called maternal instinct, but it exists (to varying degrees) in both genders.^10^
- We need to *play*. This is not as frivolous as it appears; play is the medium through which social hierarchies are formed (“pecking order”), in-group and out-group boundaries are maintained, and territory is won and defended.

Please remember: as previously stated, Panksepp (1998) distinguishes between *bodily, emotional*, and *sensory* needs, which correspond roughly with current usage of the terms “drive,” “instinct,” and “reflex.” Here I have listed only the *emotional* needs—which are felt as separation distress, rage, fear, etc.—not the *bodily* ones—which are felt as hunger, thirst, sleepiness, etc.—or *sensory* ones—which are felt as pain, disgust, surprise, etc. This focus is somewhat arbitrary, but I am highlighting the category of *emotional* needs because these most commonly give rise to psychopathology. In saying this, I do not wish to deny that *bodily* needs, too, can be enlisted in psychopathology (e.g., consider hunger in anorexia nervosa), and the same applies to *sensory* needs (e.g., consider pain in masochism). But, typically, these needs are only secondarily implicated in the psychological troubles that arise primarily from the patient's inability to meet their *emotional* needs (see next section).

I do not want to make too much of these taxonomic issues. The same applies to the disagreements between Panksepp and Ekman, say, regarding which emotions are (or are not) the truly *basic* ones. For example, Ekman considers disgust to be a basic emotion, whereas Panksepp considers it to be a *sensory* affect. (Either way, it is certainly true that disgust, like hunger and pain, can readily be enlisted in psychopathology). I say again, here we are dealing mainly with matters of principle, not with empirical details. The principle remains: *human beings—no less than other species of animal—have innate biological needs* (some of which may be described as bodily drives and some of which may be described as emotional instincts and some of which may be described as sensory reflexes). All of these needs are (ultimately) felt as effects. And all of them have to be *acted* upon. This last point leads us to the second core claim of psychoanalysis.

## Claim 2

*The main task of mental development is to learn how to meet our needs in the world*. We do not learn for its own sake; we do so in order to establish optimal *predictions* (see above) as to how we may meet our needs in a given environment. This is what Freud (1923) called “ego” development.

Learning is necessary because even innate predictions have to be reconciled with lived experience. Evolution predicts how we should behave in, say, dangerous situations in general, but it cannot predict all possible dangers; each individual has to learn *what* to fear and *how best* to respond to the variety of actual dangers they are confronted with. The most crucial lessons are learned during critical periods, mainly in early childhood, when we are—unfortunately—not best equipped to deal with the fact that our innate predictions often *conflict* with one another (e.g., attachment vs. rage, curiosity vs. fear).^11^ We therefore need to learn *compromises*, and we must find *indirect* ways of meeting our needs. This often involves *substitute-formation*. Humans also have a large capacity for *delaying* gratification and for (temporarily) satisfying their needs in *imaginary* and *symbolic* ways. This capacity is of course bound up with our large cortico-thalamic mantle, and in particular with its prefrontal component.

I now move to something fundamental. It is crucial to recognize that *successful predictions entail successful affect regulation, and vice-versa*. This is because our needs are *felt*. Thus, successful avoidance of attack reduces fear, successful reunion after separation reduces panic, etc., whereas unsuccessful attempts at avoidance or reunion result in *persistence* of the fear or panic, etc.

Please note that this formulation implies that only *unmet* needs are felt. Indeed, the meeting of a need is heralded precisely by the *disappearance* of the relevant feeling (satiation). Increasing hunger is felt as unpleasurable and decreasing hunger (relieving hunger through eating) is felt as pleasurable. These affects indicate the *direction of change* in the underlying demand (see Solms and Friston, 2018). But once the demand disappears, the feeling (both unpleasurable and pleasurable) likewise disappears. Satiation removes feelings from the radar of consciousness.

Importantly, this implies that *lack of affectivity is the ideal state of the organism*. This is what Freud (1920) called the “Nirvana principle.”^12^ We should note in passing that Freud made another important error here. He equated his Nirvana principle (i.e., aspiring to feel nothing) with a drive toward *death*. There is an inherent contradiction in the view that removing all needs (i.e., satisfying them perfectly)—which is an *ideal* biological state, the most likely to maintain and produce *life*—corresponds to a drive toward death.

This is not the place to go into all the complexities of this arcane issue. However, it seems that the source of Freud's error was his assumption that the “pleasure” and “Nirvana” principles were two *different* principles (see Solms, 2018b). Hence the phrase “*beyond* the pleasure principle” (Freud, 1920). He did not realize that feelings of pleasure and unpleasure are in fact servants of the Nirvana principle (i.e., part of the *same* principle). They merely indicate whether one is heading *further from or closer toward* the desired Nirvana (i.e., from or toward the homeostatic settling point of the need in question).

This does not mean that the clinical phenomena which Freud tried to explain with reference to a “death drive” do not exist (e.g., suicidality, anorexia nervosa, addiction, negative therapeutic reaction). It just means they are not expressions of an elemental drive. In my view the clinical phenomena in question are just that—clinical—i.e., they are *aberrations*, not biological goals. What is “deathly” about these states is their implicit failure to accept that our needs can only *really* be made to go away through *work*—i.e., through an effortful engagement *with reality*. Thus, for example, the heroin addict achieves the *illusion* of meeting their attachment needs (which are mu opioid mediated) by artificially achieving the desired affect that occurs with the presence of the caregiver without actually undertaking the work of really finding her, and what is more, without working out how to make her stay. This failure (i.e., failure to engage with the reality of the absent caregiver) is an *ego* aberration, not an id drive. Such aberrations are bound to end badly; because, in reality, we mammals need actual caregivers, not illusions of care.

Returning to the central point: *the main task of mental development is to learn how to meet our needs in the world*. As explained above, learning is necessary because even innate predictions have to be reconciled with lived experience. This is a fact. Now we can add some theory. Having established the relationship between needs and the pleasure/Nirvana principles, we may speculate (following Damasio) that learning from experience literally requires *experience*—that is, it requires *consciousness*. This statement is predicated on the above facts about the affective basis of consciousness. Conscious experience is *felt* experience. The reason why feeling must be extended outwards, onto the lived exteroceptive world, is so that the organism can determine *whether things are going better or worse there—*in the environment in which it finds itself—within our biological scale of values (in terms of which survival and reproductive success are “good” and the opposite are “bad”). As noted previously, the biological good and bad here correspond to pleasurable vs. unpleasurable feelings. In short, *exteroceptive* consciousness takes the form: I feel this *about that*.

Without feeling, therefore, there could be no *choice*. And without choice there could be no surviving in unpredicted environments, and therefore no learning from experience.^13^ *Feeling* one's way through problems (through situations not predicted by innate “survival tools”), during one's own lifetime, therefore, bestows an enormous adaptive advantage. This (feeling one's way through problems), I submit, is the essence of what we do with our “working memory.” That is what working memory is for.

Of crucial importance here is the fact that we are talking mainly about *prospective* experience. There is little biological point in learning about the likely consequences of jumping in front of a moving train by *actually trying it out*. Working memory mainly entails virtual action, not physical action. (In the life of the mind, we are—for the most part—dealing with potential energies, not kinetic energies; which has some interesting implications for the mind/body problem).

The short-term-memory process that we nowadays call working memory is what Freud called “thinking.” The essence of thinking for Freud was the fact that it is *interposed* between drives (or instincts) and action. Thinking is a process of deliberation which arises *instead of (and prior to) action*. This is crucial. This is how we supplement our innate priors (the rough-and-ready prior predictions we are born with) without actually having to commit ourselves to life-threatening courses of action, in conditions of uncertainty. This, in my view, is the only reason why cognition *needs* to become conscious. As we know, cognition typically remains unconscious (for the classical reviews, see Kihlstrom, 1996; Bargh and Chartrand, 1999). In short: our cognitions become conscious only to the extent that we need to feel them. Later we shall see that, since thinking necessarily requires inhibition of action—i.e., a *delay* function—it underwrites what Freud called the “secondary process.”

To be clear: I am not saying that thinking entails unconscious cognition plus affect (two things); I am saying it entails *conscious* cognition (one thing), which is something quite different. Through conscious cognition, raw feeling (what Friston (2010) calls variational “free energy;” see Solms and Friston, 2018) is *bound—*and this process actually changes it from the affective to the cognitive state (cf. Freud's concept of “cathexis,” which comes in two forms: bound and freely mobile). In thermodynamic terms, this (binding) means that the state of the driving energy in the mind is *transformed* through useful mental work (see Carhart-Harris and Friston, 2010).

But here comes another crucial point. Working memory (cognitive consciousness) is *a very limited resource*; so, it has to be used sparingly. This fact is well-established. It is generally referred to as Miller's law (in terms of which we are only able to hold about seven units of information in consciousness simultaneously), which in turn may be explained physiologically by way of neurotransmitter depletion.^14^ This means that the (predictive) products of thinking must be transferred from STM to LTM as rapidly as possible.^15^ In other words, to put it teleologically, STM (conscious predictive-work-in-progress) “aspires” to the LTM condition (to unconscious prediction).

This distinction between STM consciousness and LTM automatism brings to mind a famous aphorism of Freud's which may be paraphrased as “a memory trace arises instead of consciousness” (cf. Freud, 1920). The process by which this happens is, as we now know, “consolidation.” The opposite process (“consciousness arises instead of a memory trace”) is called “reconsolidation” (Nader et al., 2000; Sara, 2000; Tronson and Taylor, 2007). By “opposite process” I mean the *reversal* of consolidation; the *dissolution* of the trace: i.e., an activated trace (a salient prediction) becomes labile once more, and can therefore be revised, before it is reconsolidated.

Due to the constraints on working memory capacity just mentioned, reconsolidation is generally *resisted*. By this, I do not mean the physiological process of reconsolidation itself confronts a physiological counter-process; rather, I mean that there are biological constraints on how much uncertainty an organism can sustain. That is why roughly 95% of our goal-directed activities are executed unconsciously (Bargh and Chartrand, 1999), which means that only 5% are *not* automatized and are subject to review. To put this psychoanalytically, *the ego prefers problems to remain in the solved condition rather than the unsolved one*. Freud called this “resistance,” which gives rise to “defense.” Stated differently, and in more familiar terms: we prefer to *confirm* our predictions rather than to *disconfirm* them (cf. the “self-serving bias,” Campbell and Sedikides, 1999). Every scientist knows this bias!

The LTM predictions arising from working memory are thus stored in the corticothalamic “preconscious” and *unthinkingly* enacted, unless and until *prediction error* arises. This (prediction error, i.e., “surprise,” or falsification of the hypothesis implicit in the LTM prediction) releases “free energy” (see above). That is, surprise increases *entropy*. In terms of information theory, increased entropy implies increased *uncertainty*; and in physiological terms it implies increased *arousal* (see Pfaff, 2006; Solms and Friston, 2018). *Prediction error therefore triggers arousal, which renders the relevant preconscious prediction salient again*. It is important to notice that the “arousal” in question is not merely quantitative; as stated at the outset, it entails affective *quality*. And the quality of an affect always *means* something. Affective arousal broadcasts the presence of an *unmet need* (and the “flavor” of the affect in question identifies the *specific* need that is unmet).^16^ Stated differently: prediction error means that a prediction that was meant to meet a need did not achieve its purpose. An unmet need is thus what activates (“hypercathects,” in Freudian terms) the memory-traces that were meant to satisfy it.

On this view, only *upper brainstem and limbic arousal* can provide the activation process that is necessary for reconsolidation of a corticothalamic LTM trace to take place through working memory.^17^ (The hippocampus is, of course, part of the limbic system; it enables us to *feel* our long-term memories). For the computationally-minded, this entails the adjustment of *precision weighting* within the LTM predictive model, by the action of the core modulatory systems, which in turn—over slower time scales—drive plasticity (see Solms and Friston, 2018). Physiologically, increased precision means increased post-synaptic gain. On my view, this (precision regulation) is *the* function of the ERTAS.

So, what Friston calls *prior* predictions (what Freud called “wishes;” see below) are subjected—reluctantly—to the reality principle, whereby, through what is known as empirical Bayesian processing, they are updated (to become *posterior* predictions).

It is very important to recognize that what I have described so far involves only *cortical* memory systems. *Only cortical memory systems generate virtual realities* (consciously thinkable images, so-called “declarative” representations). These systems coincide exactly with what Freud called the “preconscious.”

Typically, the processes I have just described involve *iterative* transfers of predictive traces between three memory systems: short-term “working memory” (Freud's system Cs.) on the one hand and long-term “episodic memory” and “semantic memory” (which together constitute Freud's system Pcs.) on the other. Semantic memory is the deepest (most abstracted) of the three declarative systems.

In this respect, therefore, what Freud called “word presentations”—to the extent that language relies upon semantic memory, and vice-versa—are actually *more deeply* encoded than what he called “thing presentations” (i.e., episodic memory). Please note that “thing presentations” occur in the preconscious; they are not exclusive to the system unconscious—as even Freud (1923) himself acknowledged. However, we will have to go further than Freud on this point. Below I will claim that the unconscious (i.e., non-declarative memory) is *devoid* of “thing presentations.” On this basis I will claim that *there are no images in the unconscious* (as opposed to the preconscious). In fact, this appears to be the defining distinction between declarative and non-declarative memory. Images are the (almost) exclusive preserve of the cortex. (I say almost because crude, rough and ready “images” do exist in some brainstem structures, such as the tectum. But I use scare quotes, for the reason that these subcortical “images” never enter consciousness, which makes them curious images indeed. Who ever heard of an image that you cannot imagine?).

Now we can turn our attention to the *subcortical* memory systems.

## Claim 3

*Most of our predictions are executed unconsciously*. As we saw above, cognitive consciousness (short-term “working memory”) is an extremely limited resource, so there is enormous pressure to consolidate our solutions to life's problems into long-term memory, and then ultimately to *automatize* them. Innate predictions—of the kind discussed above^18^–are effected automatically from the outset, as are those acquired in the first 2–3 years of life, before the preconscious (“declarative”) memory systems mature (cf. infantile amnesia, which applies only to episodic and semantic memory). Multiple unconscious (non-declarative) memory systems exist, but the ones that are most relevant to psychopathology are “procedural” and “emotional” memory, which operate according to different rules. These stereotyped systems bypass thinking (cf. Freud's “repetition compulsion”) and define the mode of functioning of the system unconscious (see below).

The ultimate aim of learning is to *permanently* solve our problems (i.e., to learn how to meet our needs in the world *reliably*). To the extent that this goal is achieved, preconscious predictions are iteratively consolidated and reconsolidated *ever more deeply*. The consolidation of such *automatized* predictions centrally involves transferring them from cortical to subcortical memory systems (principally but not exclusively located in the basal ganglia and cerebellum). The crucial thing to note about these latter systems is that they entail non-representational (sometimes called “model free”) *action* programmes. Here I am using the term “representation” in the sense in which I used it above—namely to refer to *images*. That is why non-declarative memories simply cannot be retrieved into working memory; they are *non-thinkable* executive programmes.

All of this implies that truly unconscious (as opposed to preconscious) memories are *not subject to updating in working memory*. This is of crucial importance. They are, therefore, in a sense, indelible (LeDoux, 1995). But they are also highly efficient. LeDoux (1995) calls them “quick and dirty.” This is the neural basis of what Freud (1911) called the “primary process.” Via these circuits, stimulus X simply triggers response Y, with nothing in between (no delay, no thinking, no “secondary process”).^19^

This does not mean that non-declarative memories are not subject to reconsolidation. What it means is that they are not subject to reconsolidation via *thinking* (via conscious cognition, via working memory); they are only subject to reconsolidation through action. Non-declarative memories can only be activated (and thereby consolidated/reconsolidated) through embodied *enactment*.

Of course, not all automatized memories start out as declarative memories. The multiple memory systems operate both successively *and simultaneously*. Some (especially emotional memories, which arise from purely subcortical associations) are therefore automatized from the outset. This applies also to innate emotional predictions. (Instinct is just another word for innate predictions). Instinctual executive programmes are all subcortical. But—as we have seen above—they need to be *supplemented by learning*. Fear conditioning is an excellent example. Here we speak of “single-exposure learning;” e.g., we cannot afford to learn twice what happens when we stick our fingers into an electrical socket.

Learning in each of the different instinctual-emotional systems follows somewhat different rules. For example, early sexual experiences, as with fear conditioning, appear to entail single-exposure learning and to leave indelible impressions. Attachment bonds, by contrast, are established slowly during the first 6 months of life, but they become extremely difficult to change after that (cf. the difference between acute “protest” and chronic “despair” with experiences of separation and loss).

Procedural memories, similarly, are “hard to learn and hard to forget.” What these two non-declarative memory systems have in common is that *they by-pass thinking*. But this does not mean that they by-pass *affective* consciousness. Just because we cannot “declare” our automatized predictions does not mean we cannot *feel* their causes and their consequences. (The conflation of consciousness with conscious cognition—i.e., excluding affect—has often led cognitive science astray).

Now we come to the heart of the matter. I have localized Freud's system “preconscious” in the cortex and his system “unconscious” in the non-declarative memory systems located beneath the cortex, primarily in the basal ganglia, and cerebellum (Solms, 2017b). But the unconscious memory systems I have just described are conventionally called “the *cognitive* unconscious,” which is contrasted with “the *dynamic* unconscious.” Psychoanalysts acknowledge the existence of a cognitive unconscious (they call it the “unconscious ego”) but they point out that it excludes the dynamic processes that Freud discovered (which they call the “repressed”).

Freud thought the repressed unconscious was part of the id. This was one of his biggest mistakes, as I discussed above. I do not mean that the repressed unconscious does not exist. I mean only that *the system unconscious and the id are two different things*, located in two different parts of the brain.

The repressed is derived from cognitive (representational) processes, from learning, whereas the id consists in affective (non-representational) processes, and it is innate. The parts of the brain that perform the functions which Freud called “id” are located mainly in the upper brainstem and limbic system; whereas the parts that perform the functions he attributed to “the repressed” (or the “system unconscious”) are located mainly in the basal ganglia and cerebellum (There are, of course, multiple interactions between these systems. For example: the amygdala and nucleus accumbens straddle the tail and head of the caudate nucleus, respectively; and the basal ganglia, in turn, interact constantly with the prefrontal lobes).

In my opinion the difference between the cognitive and the dynamic unconscious is simply this. The cognitive unconscious consists in predictions that are *legitimately* automatized. That is, they are deeply automatized because they work so well; they reliably meet the underlying needs that they are aimed at. The repressed, by contrast, is *illegitimately* (or prematurely) automatized. Illegitimate automatization occurs *when the ego is overwhelmed by its problems*; that is, when it *cannot* work out how to satisfy id demands in the world. This happens a lot in childhood, when the ego is feeble.

The infamous Oedipus complex provides an excellent example of an insoluble problem: it is an almost-inevitable constellation of compulsive (innate) emotional needs, arising simultaneously, which are beyond the reach of the child, and irreconcilable with each other. (“Conflict” is just another word for “insoluble problem”). In such situations, *the child has no other choice but to cut its losses*. It is doomed either (1) to obsess endlessly over a problem that it cannot solve, thereby wasting precious working memory resources which could be more usefully deployed for problems that it *can* solve—such as how to read, write, and calculate—or (2) to make the best of a bad job and automatize the least-bad childish prediction it can come up with, *even though it does not work so well*.^20^

Repression (through the adjustment of precision weighting) has the inevitable implication that a deeply automatized prediction does *not* manage the feelings it is aimed at, but there is nothing the subject can do about this; since the essence of repression consists in the fact that the prediction is treated as if it *does* work well, and it is therefore *immune to reconsolidation*.^21^ The resultant prediction error is the constant pressure that Freud theorized as the threat of “the return of the repressed” (This, in turn, leads to secondary defenses—i.e., to what Freud called “after-pressure”).

Where I differ from Freud in this regard is that *I do not believe the repressed ever returns*; it is only the *affect* (which it fails to regulate) that returns. How many patients actually remember their Oedipal strivings, even in psychoanalysis, for example? This is because *non-declarative memories are just that: non-declarative*. Non-declarative memories are purely associative (and permanently unconscious) action tendencies of the kind described by LeDoux, as with Pavlov's dogs. No thinking occurs, not even implicitly. This has major implications for how psychoanalytic treatment works.

Before moving on to that topic (of how this type of treatment works), I want to restate the concluding point of this section, because it is of utmost importance: *Not only successful predictions are automatized*. With this simple observation, we have overcome the unfortunate distinction between the “cognitive” and “dynamic” unconscious. Sometimes a child has to make the best of a bad job in order to focus on the problems that it *can* solve. Such illegitimately or prematurely automatized predictions (i.e., *wishes* as opposed to realistic *solutions*) are called “the repressed.” Normally, in order for predictions to be updated in light of experience, they need to be reconsolidated; that is, they need to enter consciousness again, in order for the long-term traces to become *labile* once more. This is impossible to achieve for repressed predictions, because *the essential mechanism of repression entails immunity from (declarative) reconsolidation, despite prediction errors*.

My second argument is that the *clinical methods that psychoanalysts use to relieve mental suffering flow from the above core claims*, which are consistent with current understanding of how the brain changes. The argument unfolds over three steps:

- *Psychological patients suffer mainly from feelings*. The essential difference between psychoanalytic and psychopharmacological methods of treatment is that we believe feelings *mean something*. Specifically, feelings represent unsatisfied needs. (Thus, a patient suffering from panic is afraid of losing something, a patient suffering from rage is frustrated by something, etc.). This truism applies regardless of etiological factors; even if one person is constitutionally more fearful, say, than the next, or cognitively less capable of updating predictions, their fear still means something. To be clear: *emotional disorders entail unsuccessful attempts to satisfy needs*. That is, psychological symptoms (unlike physiological ones) involve *intentionality*.
- The main purpose of psychological treatment, then, is to *help patients learn better ways of meeting their emotional needs*. This, in turn, leads to *better emotion regulation*. The psychopharmacological approach, by contrast, suppresses unwanted feelings. We do not believe that drugs which treat feelings directly can *cure* emotional disorders; drugs are symptomatic (not causal) treatments. To cure an emotional disorder, the patient's failure to meet their underlying needs must be addressed, since this is what is *causing* the symptoms. However, symptomatic relief is sometimes necessary before patients become accessible to psychological treatment, since most forms of psychotherapy require collaborative work between patient and therapist (see below). It is also true that some patients *never* become accessible to psychotherapy. We must also concede that patients just want to feel better: they do not want to work for it.
- *Psychoanalytical* therapy differs from other forms of psychotherapy in that it *aims to change deeply automatized predictions*, which—to the extent that they are consolidated into non-declarative memory—*cannot be reconsolidated in working memory*. Non-declarative predictions are *permanently* unconscious. Psychoanalytic technique^22^ therefore focuses on:
  - Identifying the *dominant emotions* (which are consciously felt but not always recognized as arising from specific needs and their associated predictions).
  - These emotions reveal the *meaning* of the symptom. That is, they lead the way to the particular *automatized predictions* that gave rise to the symptom.
  - The pathogenic predictions *cannot be remembered directly* for the very reason that they are automatized (i.e., non-declarative). Therefore, the analyst identifies them *indirectly*, by bringing to awareness the *repetitive patterns of behavior* derived from them.
  - Reconsolidation is thus achieved through activation of non-declarative traces via their *derivatives in the present* (this is called “transference” interpretation). As stated above, non-declarative predictions cannot be retrieved into working memory; but patients *can* be made aware of the here-and-now *enactments* of those predictions. This is the essence of psychoanalytical cure.
  - Such reconsolidation is nevertheless *difficult to achieve*, mainly due to the ways in which non-declarative memory systems work (they are “hard to learn, hard to forget” and in some respects “indelible”) but also because repression entails intense resistance to the reactivation of insoluble problems (see also my comments above regarding the “self-serving bias”).
  - For all these reasons, psychoanalytic treatment takes time—i.e., *numerous and frequent sessions*—to facilitate “working through.” Working through entails numerous repetitions of transference interpretations in relation to ongoing derivates of repressed predictions, while new (and crucially, *better*) predictions are slowly consolidated.

To say the same thing in different words: repression leads to endless, mindless *repetition*; which is why “transference” is so important in psychoanalytic treatment. Patients cannot re-think the repressed (since non-declarative memories cannot be retrieved into working memory), but they can think about what they are doing now, *in consequence* of the repressed. What patients *can* think about—i.e., can re-problematize, if it is brought to their attention—are the repetitive *derivatives* of the repressed, which involve *cortical* representations (of current experiences), which can therefore enter working memory and declarative (and reflective; i.e., prefrontal) thinking. This in turn allows their (derivative) predictions to be *reconnected with the affects that belong to them*, which enables the ego to *come up with better predictions*, with more realistic action plans, with the help of an adult brain (and that of the analyst) in adult circumstances.

After transference interpretation comes the harder work of “working through,” since the establishment of new procedural memories is a *slow* process. Those who want shorter treatments, and less frequent sessions, will have to learn how non-declarative memory actually works. (Funders of psychological treatments need to learn how learning works.).

From all I have said, I hope it is clear why *our patients suffer mainly from feelings*. They don't come to us saying, “Doctor, there is something I'm unconscious of; could you please tell me what it is?” What they say is, “Doctor, I've got this [all-too-conscious] feeling that I don't want; will you please take it away.” Psychopharmacologists try to oblige patients on that score. The psychoanalytic approach, by contrast, is to help patients instead to *understand* their unwelcome feelings, i.e., to discern the errant predictions that *cause* them—i.e., the unconscious, repressed predictions—which our patients are invalidly (and unknowingly) using to meeting their emotional needs.

The analytic task is to bring these predictions back to consciousness—to re-problematize them in working memory. This is achieved by re-directing the feelings which the patient suffers from to the repressed predictions that are causing them. But, as I have said, this cannot be done *directly* in the case of non-declarative memories. It can only be done via *derivatives* of the repressed—via what is being repeated in the present moment and can therefore be “declared” and thought about. The unconscious is just that: it is *unconscious*, for ever more. Although we can infer it, we can never experience it. Such inferences (called “reconstructions” in psychoanalysis) help us to better understand the here-and-now transference. On the basis of this understanding, all that we can hope to achieve is new and better predictions; which must be *consolidated alongside the old ones*.^23^ But since the new ones are better at meeting the underlying needs, they are (gradually) deployed more readily by the patient, and thus consolidated, ever more deeply, even after the treatment ends. This last point explains the well-established “sleeper effect,” whereby symptoms continue to improve after the termination of psychoanalytic treatments (see below).

There are many other things I would have liked to discuss here, such as how we use affects in the so-called “countertransference;” but that is not my focus in this article (for a more clinically oriented discussion, see Smith and Solms, 2018).

My third and final argument is that *psychoanalytic therapy achieves good outcomes*—at least as good as, and in some respects better than, other evidence-based treatments in psychiatry today (see Shedler, 2015). This argument unfolds over four stages:

- *Psychotherapy in general is a highly effective form of treatment*. Meta-analyses of psychotherapy outcome studies typically reveal effect sizes of between 0.73 and 0.85. (An effect size of 1.0 means that the average treated patient is one standard deviation healthier than the average untreated patient). An effect size of 0.8 is considered a large effect in psychiatric research, 0.5 is considered moderate, and 0.2 is considered small. To put the efficacy of psychotherapy into perspective, recent antidepressant medications achieve effect sizes of between 0.24 (tricyclics) and 0.31 (SSRIs).^24^ The changes brought about by psychotherapy, no less than drug therapy, are of course visualizable with brain imaging (see Beauregard, 2014).
- *Psychoanalytic psychotherapy is equally effective* as other forms of psychotherapy (e.g., CBT). This has recently been demonstrated conclusively by comparative meta-analysis (Steinert et al., 2017). However, there is evidence to suggest that *the effects last longer—and even increase—after the end of the treatment*. (Shedler, 2010) authoritative review of all randomized control trials to date reported effect sizes of between 0.78 and 1.46, even for diluted and truncated forms of psychoanalytic therapy.^25^ An especially methodologically rigorous meta-analysis (Abbass et al., 2006) yielded an overall effect size of 0.97 for general symptom improvement with psychoanalytic therapy. The effect size increased to 1.51 when the patients were assessed at follow-up. A more recent meta-analysis by Abbass et al. (2014) yielded an overall effect size of 0.71 and the finding of maintained and increased effects at follow-up was reconfirmed.
  This was for *short-term* psychoanalytic treatment. According to the meta-analysis of de Maat et al. (2009), which was less methodologically rigorous than the Abbass studies, *longer-term* psychoanalytic psychotherapy yields an effect size of 0.78 at termination and 0.94 at follow-up, and *psychoanalysis* proper achieves a mean effect size of 0.87 at termination and 1.18 at follow-up. This is the overall effect; the effect size that she found for symptom improvement (as opposed to personality change) at termination was 1.03 for long-term therapy, and for psychoanalysis it was 1.38. A subsequent study by Leuzinger-Bohleber et al. (2018, in press) shows even bigger effect sizes: between 1.62 and 1.89 after 3 years of treatment. These are enormous effects. Follow-up data are of course not yet available from this ongoing study. The consistent trend toward *larger effect sizes at follow-up* (where the effects of other forms of psychotherapy, like CBT, tend to *decay*) suggests that psychoanalytic therapy sets in motion processes of change that continue even after therapy has ended (cf. “working through,” discussed above). This is called the “sleeper effect.”
  It is important to recognize that these findings concern symptom improvement only. Psychoanalytic treatments are not directed primarily at symptomatic relief but rather at what might be called personality change. Not surprisingly, therefore, psychoanalytic treatments achieve much better results than other treatments on *this* outcome measure. In Leuzinger et al.'s ongoing study, for example, almost twice as many patients receiving psychoanalytic treatment vs. CBT reached their criteria for “structural change” after 3 years (60 vs. 36%).
- The therapeutic techniques that predict best treatment outcomes *make good sense in relation to the psychodynamic mechanisms outlined above*. These techniques are (Blagys and Hilsenroth, 2000):
  - *unstructured*, open-ended dialogue between patient and therapist.
  - identifying *recurring themes* in the patient's experience.
  - linking the patient's *feelings* and perceptions to *past experiences*.
  - drawing attention to *feelings* regarded by the patient as *unacceptable*.
  - pointing out ways in which the patient *avoids* feelings.
  - focusing on the here-and-now therapy relationship.
  - drawing connections between the therapy relationship and other relationships.
  It is highly instructive to note that these techniques lead to the best treatment outcomes, *regardless of the “brand” of therapy the clinician espouses*. In other words, these same techniques (or at least a subset of them; see Hayes et al., 1996) predict optimal treatment outcomes in CBT too, even if the therapist believes they are doing something else.
- It is therefore perhaps not surprising that psychotherapists, irrespective of their stated theoretical orientation, tend to choose psychoanalytic psychotherapy for themselves (Norcross, 2005)!

## Conclusion

I am aware that the neurobiological assumptions and hypotheses outlined in this article are synthesized in a highly abstracted way. My aim has been only to sketch the bigger picture, in broad brushstrokes, so that the wood emerges from the trees. I hope that this rough sketch has served its essential purpose, which is to provide in simple terms a neurobiological understanding of psychoanalytic theory and therapy, as things stand today. I do not mean to assert, of course, that psychoanalysis was *based upon* these underpinnings. Rather, I hope to have shown that the core theoretical claims and technical practices of psychoanalysis have gradually *acquired* neurobiological support.

I am also well aware that the claims I have summarized here do not do justice to the full complexity and variety of views in psychoanalysis, both as a theory and a therapy. I am saying only that these are the *core* claims, which underpin all the details, including some of those upon which psychoanalysts are yet to reach agreement. I believe that these claims are increasingly supportable, in light of current scientific evidence, and that they make simple good sense.

## Author Contributions

The author confirms being the sole contributor of this work and has approved it for publication.

### Conflict of Interest Statement

The author declares that the research was conducted in the absence of any commercial or financial relationships that could be construed as a potential conflict of interest.

## Footnotes

^1^Before readers exclaim “who ever doubted *that*?”, let us recall: academic psychology departments were dominated during much of the twentieth century by a theory which questioned precisely that. The rival theory was called “behaviorism.” The Wikipedia entry for “instinct,” for example, states that “Instinct as a concept fell out of favor in the 1920s with the rise of behaviorism and such thinkers as B. F. Skinner, which held that most significant behavior is learned.”

^2^Freud's localization of consciousness underwent many vicissitudes. Initially he made no distinction between perceptual and affective consciousness (Freud, 1894). Rather he distinguished between *memory traces of perception* (“ideas”) and the *energy that activates them*. This distinction coincided with the conventional assumptions of British empiricist philosophy, but Freud interestingly described the activating energy as “quotas of affect,” which are “spread over the memory-traces of ideas somewhat as an electric charge is spread over the surface of a body” (Freud, 1894, p. 60). Strachey (1962, p. 63) described this as the “most fundamental of all [Freud's] hypotheses.” There is every reason to believe that Freud envisaged such activated memory traces of “ideas” as *cortical* processes. In his more elaborated Freud (1950 [1895-96]) “Project” model, he explicitly attributed consciousness to a subsystem of cortical neurons (the ω system), which he located at the *motor* end of the forebrain. This location enabled consciousness to register discharge (or lack thereof) of the energy that accumulated over the memory traces (the ψ system) from both endogenous and sensory sources (Please note: from 1895 onward Freud described mental energy as being unconscious in itself; it was no longer described as a “quota of affect”). Consciousness, which Freud now divided into two forms, arose from the manner in which mental energy excited the ω neurons. It gave rise to *affective* consciousness when differences in the quantitative level of energy in the ψ system (caused by degrees of motor discharge) was registered in ω as pleasure-unpleasure; and it gave rise to *perceptual* consciousness when differences in qualitative aspects of exogenous energies (e.g., wavelength or frequency) derived from the different sense organs were transmitted, via perceptual (ϕ) neurons, through the memory traces of ideas (ψ), onto ω. In an 1896 revision of this “Project” model, Freud moved the ω neurons to a position between ϕ and ψ, and simultaneously acknowledged that all energy in the mental apparatus was endogenously generated; energy did not literally enter the apparatus through the perceptual system. (Freud seemed to forget this later; e.g., 1920.) In *The Interpretation of Dreams* 1900, however, Freud reverted to the “Project” arrangement, and again located the perceptual and consciousness systems at opposite ends of the mental apparatus. His indecision in this respect seems to have derived mainly from the fact that the cortical perceptual (sensory) and consciousness (motor) systems form an integrated functional unit, since motor discharge necessarily produces perceptual information (Cf. the contiguous location of the somatosensory and motor homunculi). Freud accordingly settled (in 1917) on a hybrid localization of the perceptual and consciousness systems. In this final arrangement, ϕ (renamed “Pcpt” in 1900) and ω (‘Cs’) were combined into a single functional unit, the system “Pcpt-Cs.” At this point Freud clarified that the Pcpt-Cs system is really a single system which is *excitable from two directions*: exogenous stimuli generate perceptual consciousness, endogenous stimuli generate affective consciousness. Freud also retreated from the notion that affective consciousness registers the quantitative “level” of excitation within the ψ system, and suggested instead that it—like perceptual consciousness—registers something qualitative, like wavelength (i.e., fluctuations in the level of energy within the Pcs system over a unit of time; see Freud, 1920). The main thing to notice in this brief history of Freud's localization of consciousness is that it was from first to last conceptualized as a *cortical* process (Although Freud did seem to have fleeting doubts about this at times; e.g., 1923, p. 21). See (Freud, 1940) (quoted in the text below) for explicit confirmation that his cortical localization of consciousness applied to both perceptual *and* affective consciousness. See Solms (1997) for a first intimation that something was wrong with Freud's superficial localization of the internal (affective) surface of the system Pcpt-Cs.

^3^If affective consciousness truly was a property of cortex, Freud's “pleasure principle” would be a top-down regulatory principle, which it is not (see e.g., Freud, 1911).

^4^Cf. Freud's description of the process: “Cathectic innervations are sent out and withdrawn in rapid periodic impulses from within [the id] into the completely pervious system *Pcpt.-Cs*. So long as that system is cathected in this manner it receives perceptions (which are accompanied by consciousness) and passes the excitation onwards to the unconscious mnemic systems; but as soon as the cathexis is withdrawn, consciousness is extinguished and the functioning of the system comes to a standstill. It is as though the unconscious stretches out feelers, through the medium of the system *Pcpt.-Cs*., toward the external world and hastily withdraws them as soon as they have sampled the excitations coming from it” (Freud, 1925, p. 231). Note that Freud's “feelers” are *unconscious* until they reach the cortical system *Pcpt.-Cs*. To reconcile his conception with contemporary knowledge, we should say “the id [not the unconscious] stretches out feelers.”

^5^The taxonomy of innate needs is an empirical question, of the kind I mentioned earlier; it does not alter the basic claim that *we are born with a set of innate needs*, which are felt as affects and which trigger stereotyped predictions. I am well aware that the taxonomy I cite here differs from Freud's. Unlike many of his followers, Freud (1920) accepted that biology might well “blow away the artificial fabric of our hypotheses [about the nature and number of instincts].”

^6^The fact that we can only meet our needs by engaging with others is why life is difficult. You cannot successfully copulate with yourself, attach to yourself, etc., although this does not stop us from trying (The psychoanalytic theory of “narcissism” arises from these simple facts)!

^7^For this easily-understandable reason, Freud conflated them.

^8^The relationship between (fear) anxiety and libido has a long history in Freud's work. Suffice it to say that they—like all of the instincts enumerated here—turn out to have distinctly separable brain circuits and chemistries.

^9^Here too, the evidence ultimately favored those psychoanalysts (like Fairbairn and Bowlby) who asserted that attachment and lust are two independent biological needs.

^10^Notwithstanding what I say above, it is interesting how closely intertwined is this brain system with the circuitry for female lust.

^11^This is why childhood, and the quality of parental guidance, are so important in psychoanalysis.

^12^Which can in turn be traced back to his “principle of neuronal inertia” (Freud, 1950 [1895-96]).

^13^This type of learning literally saves lives. The alternative is learning through natural selection, over generations; i.e., what works was selected (and became an innate prediction) because it facilitated the survival and therefore reproductive success of our ancestors. Pity about all the others, who made the wrong random “choices.”

^14^STM traces decay rapidly as a consequence of neurotransmitter reuptake mechanisms that restore presynaptic neurons to the state that existed prior to the formation of each trace; thereby enabling them rapidly to form further traces. See Mongillo et al. (2008).

^15^Another, closely related, reason for this is that a complex organism has to *set priorities*. In order to determine “what to do next,” problems must be prioritized. This is because, generally, it is not possible to do two things at once (e.g., one must eat first, drink second; defeat the rival first, copulate second). The capacities of the motor system, no less than those of working memory, establish an executive “bottleneck” (see Merker, 2007).

^16^I am of the view that this “flavoring” (or “color coding”) of different needs via affect is an important facilitator of the *prioritizing* processes discussed above. It enables the brain to identify and compartmentalize computations requiring updating from those that do not, and thus to reduce computational complexity and save on processing power. This is an important part of the causal contribution of qualia to neural information processing (see Solms and Friston, 2018).

^17^See Puryear and Mizumori (2008). Cf. the “global workspace” theory of consciousness. See also Haubrich et al. (2015).

^18^Please note: there probably are no innate *cortical* predictions. See Ellis and Solms (2018).

^19^See footnote below for further discussion of what Freud (1915b) called “the special characteristics of the system Ucs.”

^20^Please note: on this view, the Oedipus complex is derived from experience (Here I am differentiating the Oedipus complex itself from the needs which give rise to it).

^21^This is why the system Ucs is *timeless*. This is also why it *tolerates mutual contradiction*; which simply means that it tolerates unsolved problems. The same applies to the another “special characteristic” of the system Ucs, namely its preference of *psychical vs. material reality*; which simply means that it is impervious to evidence. The fourth special characteristic of the Ucs is *primary process* mobility of cathexes, which I have already discussed in the text above.

^22^See Blagys and Hilsenroth (2000) and Smith and Solms (2018).

^23^The persistence of the old predictions is why patients can sometimes get worse (regress) during times of stress—revert to their old ways.

^24^See Turner et al. (2008) and Kirsch et al. (2008).

^25^I would like to thank Jonathan Shedler for his generous help with this section of my paper, which is based on Solms (2018a).
